# Comparison and Analysis of Membrane Fouling between Flocculent Sludge Membrane Bioreactor and Granular Sludge Membrane Bioreactor

**DOI:** 10.1371/journal.pone.0040819

**Published:** 2012-07-30

**Authors:** Wang Jing-Feng, Qiu Zhi-Gang, Chen Zhi-Qiang, Li Jun-Wen, Zhang Yi-Hong, Wang Xuan, Zhang Bin

**Affiliations:** 1 Institute of Health and Environment Medicine, Academy of Military Medicine Science, Tianjin, China; 2 Tianjin Key Laboratory of Risk Assessment and Control for Environment & Food Safety, Tianjin, China; 3 Tianjin Key Laboratory of Hollow Fiber Membrane Material and Membrane Process, Institute of Biological and Chemical Engineering, Tianjin Polytechnical University, Tianjin, China; Loyola University Medical Center, United States of America

## Abstract

The goal of this study is to investigate the effect of inoculating granules on reducing membrane fouling. In order to evaluate the differences in performance between flocculent sludge and aerobic granular sludge in membrane reactors (MBRs), two reactors were run in parallel and various parameters related to membrane fouling were measured. The results indicated that specific resistance to the fouling layer was five times greater than that of mixed liquor sludge in the granular MBR. The floc sludge more easily formed a compact layer on the membrane surface, and increased membrane resistance. Specifically, the floc sludge had a higher moisture content, extracellular polymeric substances concentration, and negative surface charge. In contrast, aerobic granules could improve structural integrity and strength, which contributed to the preferable permeate performance. Therefore, inoculating aerobic granules in a MBR presents an effective method of reducing the membrane fouling associated with floc sludge the perspective of from the morphological characteristics of microbial aggregates.

## Introduction

The membrane bioreactor (MBR) is an efficient and compact processing technology that has been widely studied and applied in wastewater treatment and reuse [1], [2]. Because the activated sludge in reactor cannot pass through the membrane, substances accumulate on the membrane surface and/or clog the actual filter pores, resulting in membrane fouling and an increase in filtration resistance. While effective in many wastewater treatment scenarios, membrane fouling is a recurring problem that has limited further development and application of MBRs [1]–[4]. To minimize the membrane fouling problem, a MBR is either run at critical permeate flux, which optimizes the aeration intensity to remove membrane particulates, or is frequently cleaned by physical or chemical methods [2], [5]. Both of these procedures are time-consuming and add to the fundamental processing costs; therefore, a more effective solution would be welcomed by wastewater engineers and plant operators. Previous studies have identified sludge concentration as a key factor contributing to membrane fouling [6]. However, subsequent studies have shown that there are several sludge characteristics in addition to concentration that impact membrane fouling, including floc size, liquid viscosity, microbial extracellular polymeric substances (EPS) and soluble microbial products (SMP). Not surprisingly, more recent studies have focused on development of effective modifications that would improve sludge performance to reduce membrane fouling. One possible improvement is to extend the life of membrane modules by adding adsorbent (e.g., activated carbon, organic polymers) into the reactor. The results of previous studies suggest that additional adsorbent could improve sludge floc structure, reduce the accumulation of organic matter in the suspended liquid and thus delay membrane fouling [7], [8]. In other studies, membrane fouling was found to be reduced by adding coagulant to the MBR [9], [10].

Although adding exogenous substances can reduce fouling to some extent, it also adds foreign materials to the reactor, and may reduce the sludge activity. In addition, these methods often fail to produce a sustainable effect without regular re-dosing. Therefore, a more effective means of optimizing filtration performance may be the key to resolving the membrane fouling problem. Currently, flocculent sludge is used in almost all membrane bioreactors. However, as an alternative, aerobic granular sludge may be more appropriate than floc sludge for MBRs [11]–[13]. Aerobic granular sludge has a relatively larger size and dense structure [14]–[17]. Additionally, its settling performance and filtering capabilities are superior to floc sludge [18]–[20]; therefore, it has the potential to reduce membrane fouling. While granular sludge is not new, there has been a recent upsurge of interest in aerobic granulation owing to its high reactivity and the multiple-function micro-environment formed by its unique spatial dimensions [21], [22]. In this study, the two MBRs were operated in parallel, which were inoculated with aerobic granular sludge and floc sludge respectively, and changes in the permeate flux were monitored. To quantitatively evaluate the differences between the two systems with respect to filtration performance and membrane fouling, the filtration resistance distribution of the membrane module in the granular sludge MBR was analyzed and a simultaneous comparison of sludge size distribution, specific resistance and EPS was conducted.

## Results

### Membrane Performance in SMBR and GMBR

**Figure 1:**
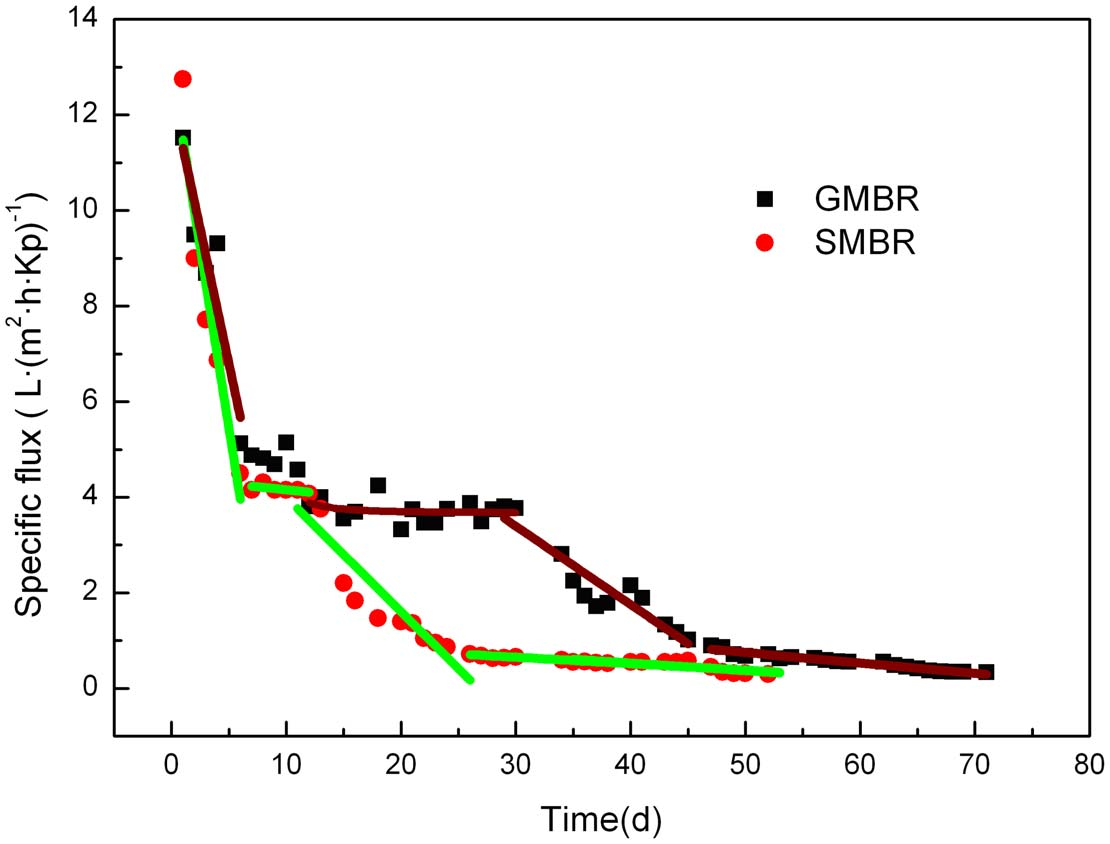
Changes in membrane specific permeate flux in both MBRs.

During the study the SMBR and GMBR were operated continuously, and the membranes were not hydraulically or chemically cleaned. As illustrated in Fig. 1, the membrane flux differed between the two reactors over the operational period. Based on the quantitative measurements, the changes in permeate flux were divided into four stages: initial rapid decline, stable, slow decline and operation (characterized by low flux). The response of both reactors was similar in the initial 6d of operation, during which time there was a rapid decline of flux, culminating in a relatively short, stable flux phase at about 4 l·(m^2^·h·kp)^−1^. The GMBR remained stable for a longer period of time, beginning a second decline at about 30 d. On the other hand, the SMBR began a second decline in membrane flux at about 12 d. By day 45, the GMBR had ceased its decline in flux and once again stabilized. The drop in SMBR flux stopped at about day 22, after which a stable flux was observed. Operation of the SMBR was terminated at 53 d because its membrane flux had dropped to less than 0.3 l·(m^2^·h·kp) ^−1^. However, membrane flux of the GMBR remained at 0.33 l·(m^2^·h·kp) ^−1^ when operations were halted at 71 d. This difference was a strong indicator of the superior membrane performance and longer membrane life of the GMBR.

### Effects of Sludge Morphology and Particle Size

Filter performance of sludge is closely associated with sludge particle morphology and chemical/physical characteristics, and sludge filtration performance directly affects the transmembrane pressure (TMP). While the SMBR sludge was always present in floc form, some of the incubated aerobic granular sludge in the GMBR broke apart in association with new bacterial micelle growth, resulting in a combination of granular sludge and floc sludge in the reactor.

**Figure 2:**
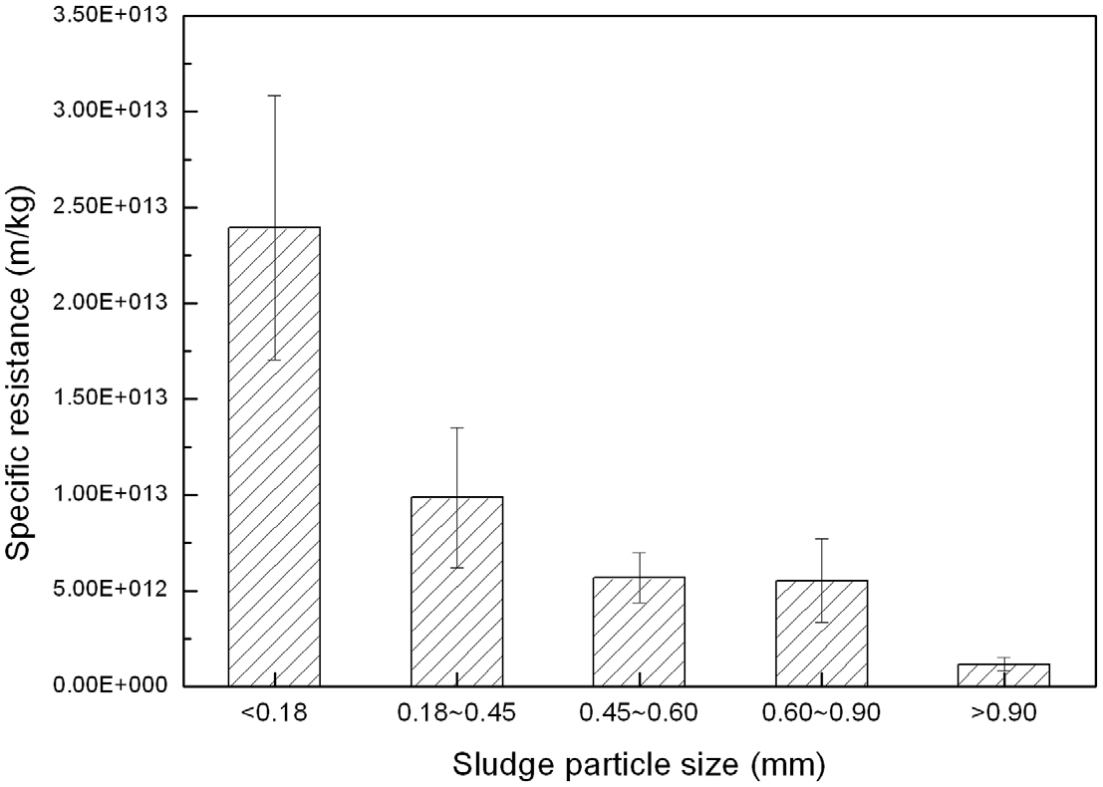
Specific resistance of different-size sludge particles in the GMBR at the steady flux stage.

Sludge from the GMBR was selected for further investigation of the filtration performance of the different sludge forms. The mixed sludge from the steady flux stage of the GMBR operation was screened, after which the resistance was analyzed as a quantitative measure of filtration performance. The sludge which particle diameter (*d*) was smaller than 0.18 mm was defined as floc sludge, whereas the other sludge ranges (*d* >0.18 mm) were regarded as granules. The resistance of floc sludge was found to be significantly higher than that of the aerobic granular sludge (Fig. 2). The resistance of the aerobic granular sludge decreased with increasing particle size. The average floc sludge resistance was measured to be 1.8×10^13^ m/kg, which resulting in poor filter performance. And the value was 2.4, 4.2, 4.3 and 20.6 times that of the granular sludge in the 0.18–0.45, 0.45–0.60, 0.60–0.90 and >0.90 mm size ranges, respectively.

### Particle Size Distribution in the Contamination Layer

**Figure 3:**
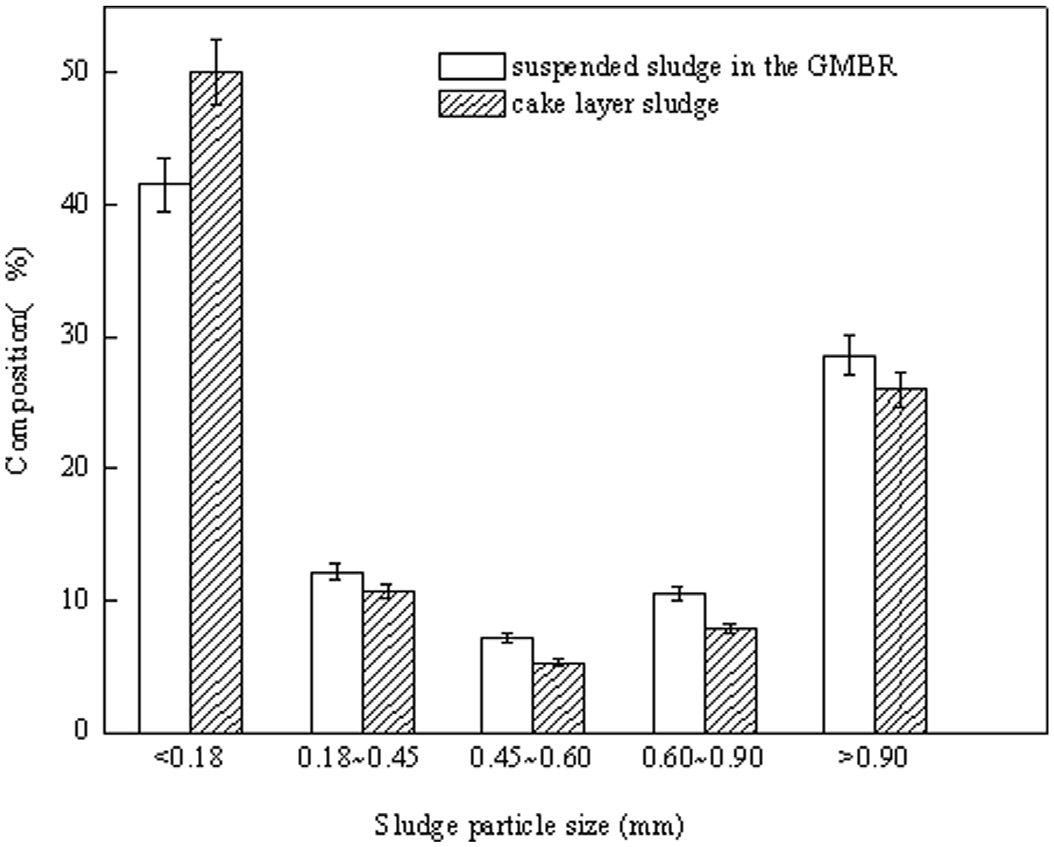
Size distribution particles in the contaminated layer of sludge and the mixed liquor sludge in the GMBR.

Sludge from the contamination layer was removed on day 71, and particle size distribution was shown in Fig. 3. The sludge was dominated by flocculent sludge range (<0.18 mm), which accounted for 50% of the total sludge in the contamination layer. Whereas, proportion of the suspended sludge less than 0.18 mm was 42%. The proportions of granular sludge of various sizes in the contamination layer were all slightly lower than that in the suspended sludge. These data indicate that a large amount of the granular sludge tends to remain suspended rather than settle on the membrane surface. Conversely, the majority of floc sludge settles on the membrane surface, exacerbating the density of the sludge layer, increasing the TMP and promoting more rapid fouling.

### Filtration Performance of Membrane Fouling Layer

**Figure 4:**
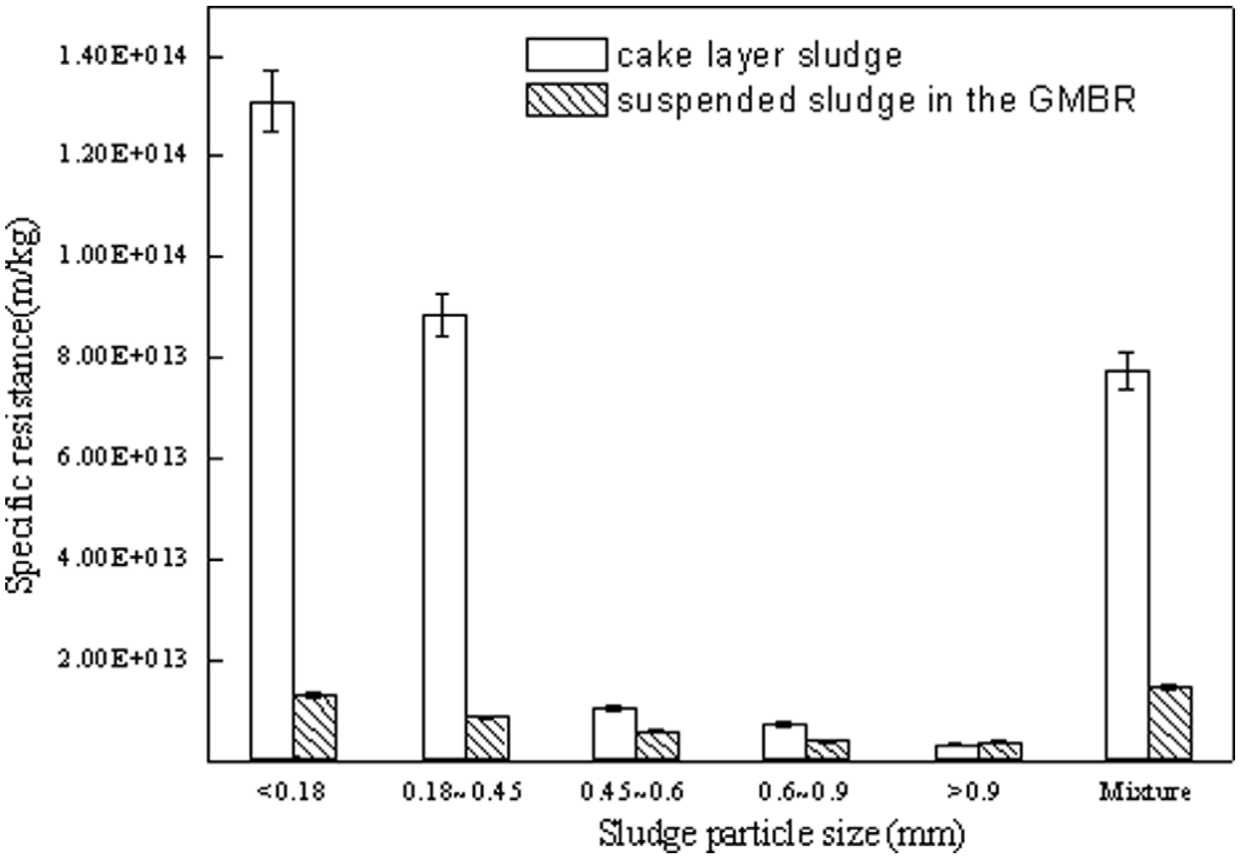
Specific resistance of the contaminated layer of sludge and the mixed liquor sludge in the GMBR.

The filtration performance of sludge in a MBR and, in particular, the sludge contaminated layer, is the key to stable and efficient operation. The specific resistance of different size granules within the fouling layer on the GMBR membrane surface are shown in Fig. 4. When compared with the filtering performance of the mixed suspended sludge, the average resistance of mixed sludge in the membrane fouling layer was 7.76×10^13^ m/kg, which was 5.28 times that of the mixed suspended sludge. This difference suggested worse filter performance of contaminated layer, relative to the mixed suspended sludge. The resistance of the flocculent sludge (<0.18 mm) in the contaminated layer reached a maximum of 1.31×10^14^ m/kg, which was about 10.2 times that of the flocculent sludge in the suspended sludge (Figure 4). The specific resistance of the layer containing granular sludge particles in the 0.18–0.45 mm range was 8.85×10^13^ m/kg, which was also 10.2 higher than the same size range of suspended sludge. As the sludge particle size rose, particularly when it was above 0.45 mm, the resistance of the granular sludge on the contaminated membrane surface increased only slightly above that of the suspended sludge. Within each particle size range, the resistance of the suspended sludge was less than that of the sludge of the contaminated layer (Fig. 4), thus enhancing filter performance.

In the membrane surface contaminated layer, the resistance of sludge particles in the 0.18–0.45 mm range was much higher than the resistance of large granules (>0.45 mm). Therefore, the size of the particles plays a key role in membrane filtration capability, with <0.45 mm particles making a substantial contribution to the sludge filter cake layer and decreasing the performance of the membrane.

### Characteristics of Sludge on the Membrane Surface

Because the traits of the contaminated layer sludge are more indicative of the fouling process than those of the suspended sludge, the former from the GMBR were selected for a more detailed evaluation of its different size fractions (Table 1). There were substantial differences between floc sludge and granules in the contaminated layer on the membrane surface. The floc sludge (<0.18 mm) had a higher moisture content and a much higher EPS concentration that was over twice that of the higher granular particle size groups(d>0.6 mm). In addition, the surface of the smallest particles was more negatively charged than that of the larger materials. These characteristics tend to promote surface accumulation of sludge materials, thus reducing filter performance. These data are consistent with information showing that the floc sludge had higher resistance (Fig. 4). In contrast, characteristics of the granular sludge promoted improved sludge filter performance and slowing of the fouling process.

**Table 1:** Characteristics of different sludge size fractions within the contaminated layer on the membrane surface^a^.

Sludge size (mm)	Moisture content (%)	VSS/MLSS (%)	EPS (mg/L)	Sludge surface charge (meq/L)
**<0.18**	99.85±0.04	94.55±0.63	198.20±3.07	^−^0.957±0.029
**0.18–0.45**	99.36±0.05	95.10±0.24	110.13±1.59	^−^0.831±0.028
**0.45–0.60**	98.73±0.09	96.10±0.23	111.18±2.30	^−^0.676±0.036
**0.60–0.90**	98.61±0.12	96.55±0.27	87.53±0.62	^−^0.326±0.031
**>0.90**	97.91±0.06	96.68±0.19	96.54±1.62	^−^0.252±0.030

a Values are 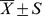 (*n* = 3).

Because the water content of granular sludge particles >0.45 mm was lower than that of the floc sludge, the volume of the granular sludge could be reduced by 50% or more on a dry sludge weight basis; therefore, the thickness of the sludge cake layer that forms during the filtration process is also lower. A thinner cake layer translates to faster filtration and reduced TMP. Lower EPS concentrations also reduce fouling. In fact, EPS concentration and composition have been implicated as controlling factors of membrane fouling. The type of molecules (e.g., proteins, carbohydrates) that comprise EPS can have a significant impact on particle surface charge, making it more negative. This, in turn, attracts positively-charged suspended materials, causing them to settle out of solution and add to the filtration resistance. The VSS/MLSS also differed among particle size classes, with the lowest value being observed for the floc sludge. This was likely because the floc sludge has smaller and more variable particles and can therefore directly cover the membrane surface in a more dense cake layer with relatively few interstitial spaces for permeation of fluid. Conversely, the contaminated layer formed from the larger granular sludge particles is less intense and exhibits larger interstitial voids, which is favorable to the filtering process.

## Discussion

### Advantage of Granular Sludge

Sludge filter performance is intimately linked to the nature of the mixture, with the primary driver of filtration resistance being sludge particle size. When the sludge particle size is smaller, the rate of net migration to the filter surface area is greater, and the sludge is more easily deposited on the membrane surface, forming a compact, less permeable, sedimentary layer [23].

Flocculent sludge is often characterized by a small size, with a loose, unconsolidated structure, and high moisture content. These characteristics promote rapid deposition on the membrane surface. All sludges in MBRs undergo certain morphological alterations during the operational period, resulting in dense sedimentation and an inevitable increase in filtration resistance. However, aerobic granular sludge is not only larger, but also relatively dense [24]. As such, it can maintain a relatively stable form throughout the filtration process, thereby reducing filtration resistance.

The improved stability and lower sedimentary characteristics of granular sludge enable it to facilitate favorable and longer-lasting flux conditions in a MBR. To support these improved reactor characteristics, the quantity of aerobic granular sludge and its larger size must be maintained. In this study, larger-diameter aerobic granular sludge underwent a certain level of disintegration. In the later operational stages, the concentration of granular sludge particles that were larger than 0.9 mm was only 45% of that at the time of system inoculation, while the concentration of smaller particles rose. Overall, the proportion of granular sludge in the test reactor remained between 52% and 60% of the total sludge concentration. Additional strategies for maintaining granular sludge levels and composition near the initial values should be focused in future investigations.

### The Effect of Membrane Surface Sludge Contaminated Layer on Membrane Fouling

The fouling layer on the membrane surface was the primary cause of higher TMP. Because effluent must pass through the layer before contacting the membrane surface, its characteristics have a direct impact on filtering capacity and reactor efficiency [25]. Studies have shown that the proportion of floc sludge in the contaminated layer was higher than that in suspended sludge (Fig. 3). This difference is primarily related to the greater tendency of floc sludge to attach to and accumulate at the membrane surface as it is carried by directional effluent flow through the system. Conversely, granular sludge has larger particles and a more consolidated structure [24]; therefore, it tends to remain in suspension and is less vulnerable to accumulation on the membrane. The accumulation of floc sludge on membrane surface changes the composition of the contaminated layer, thus increasing the specific resistance (Fig. 4).

EPS is one of the sludge metabolites that exacerbated the fouling problem, which accumulated on the membrane. Once a significant amount of contaminated layer is formed, it also contributes to the resistant force that the filtrate must overcome to pass through the membrane. EPS within the contaminated layer of sludge will continuously fill in available gaps, thus elevating filtration resistance and reducing system efficiency. Another aspect of EPS that contributes to increased system inefficiency is its composition [25]. Proportionally, protein is the largest component of EPS, followed by humus and carbohydrates. However, carbohydrates in the EPS show the most significant increase in the contaminated layer sludge, being as much as 2.64 times that of suspended sludge (Table 1). Since carbohydrates tend to be hydrophilic, an increase in carbohydrates will cause a concomitant increase in the moisture content of the sludge. This, in turn, exacerbates the problem of reduced filtering performance.

In Conclusion, the filtering performance of aerobic granular sludge was superior to that of flocculent sludge. Comparison of the performance of continuously-operating membranes in the SMBR and GMBR revealed that inoculation of the MBR with aerobic granular sludge effectively slowed the process of membrane fouling and extended membrane life. Significantly different characteristics were identified among the forms and size groups of sludge in the membrane surface contamination layer. Floc sludge had a high water content, EPS concentration and negative surface charge, which are important factors contributing to membrane fouling and poor sludge filter performance.

## Materials and Methods

### Experimental Design and Method of Operation

**Figure 5:**
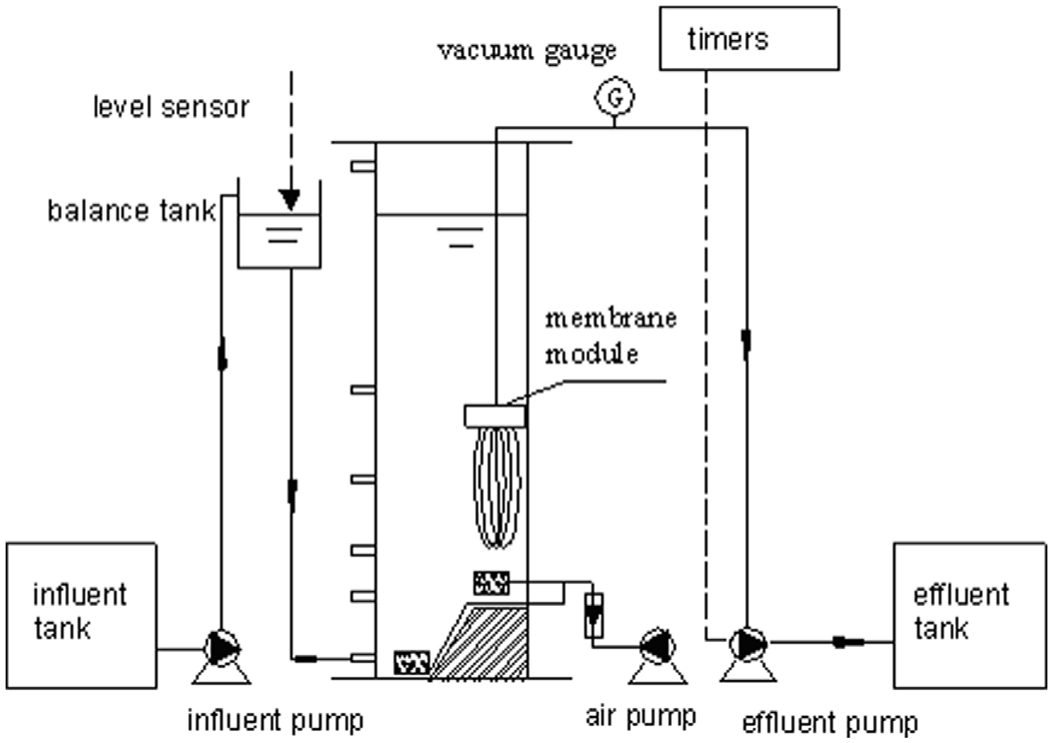
Schematic diagram of the MBR system.

Two separate MBRs were inoculated with either flocculent sludge or aerobic granular sludge and marked as SMBR (sludge MBR) and GMBR (granule MBR), respectively (Fig. 5). The working volume of each reactor was 7.6 l. A U-shape polyvinylidene fluoride (PVDF) hollow fiber membrane module with a pore size of 0.22 µm and total membrane surface area of 0.3 m^2^ was submerged in both reactors. Air was supplied from microporous air diffusers at a gas:water ratio of 30–50∶1. The air demand was controlled by a pneumatic rotameter. The effluent was removed by a peristaltic pump by applying intermittent suction at an 8 min/2 min interval.

### Wastewater Composition and Seeding Sludge

The components of the synthetic influent wastewater were as follows: sodium acetate (as an organic source) 150–400 mg/l; NH_4_Cl-N 25-40 mg/l; KH_2_PO_4_-P 2-8 mg/l; MgSO_4_·7H_2_O 50 mg/l; CaCl_2_ 20 mg/l; KCl 20 mg/l; MnCl_2_ 0.1 mg/l; FeSO_4_·7H_2_O 0.1 mg/l; CuSO_4_·5H_2_O 0.1 mg/l; ZnSO_4_ 0.1 mg/l.

The flocculent sludge inoculated into the SMBR was obtained from a secondary sedimentation tank at a local sewage treatment plant. The average particle size (D[4], [3]) of the sludge was 0.082 mm, with a sludge volume index (SVI) of 80–120. The aerobic granular sludge inoculated into the GMBR was cultured in the laboratory [26], and 88% of the sludge granules were >0.9 mm. The granules were grey black with an appearance of fine sand. The actual granule size ranged from 0.8 to approximately 1.5 mm, with the SVI of 30–50.

### Analytical Methods

The separation and distribution of different size granules was determined by the sieving method [27]. To accomplish this, 100 ml of sludge in the reactor was collected using a calibrated cylinder. The samples were then screened through four stainless steel sieves with a 5 cm diameter that had mesh openings of 0.18–0.9 mm. Sludge was separated into five particle size (<0.18 mm, 0.18–0.45 mm, 0.45–0.6 mm, 0.6–0.9 mm and >0.9 mm) by screening the sludge through different sized sieves. The granules retained on the different screens were then recovered by a backwash using distilled water and collected in a beaker for further analysis. At the end of experiment, the membrane module was pulled out of the reactor. The fouling module was rinsed with pure water; meanwhile the attached cake layer on the membrane was taken out of the module physically using hands. These sludge were regarded as contaminated lay. The different sized granules in the contaminated layer were then separated according to the steps described above. Although there is no clearly defined size that separates flocculent and granular sludge particles, 0.18 mm was selected as a reasonable boundary between flocculent sludge (<0.18 mm) and granular sludge (≥0.18 mm) for this study. The specific resistance to filtration (SRF) was determined using a pressurized terminal filtration device modified from a stirred ultrafiltration cell(MSC300, Mosu Instruments Co., Ltd., Shanghai, China) at 138kPa [28], the average pore size of flat membrane filters (cellulose nitrate and cellulose acetate polymers) was 0.22 µm. This combination of the filtration apparatus and filters was shown to effectively retain all dispersed microorganisms, yielding SRF values that better reflect actual biomass dewaterability. The specific gravity and the kinematic viscosity of the filtrate were determined using a 50 ml Gay-Lussac specific gravity bottles (Tianbo Company, Tianjin, China) and a calibrated viscometer (DV-3+PRO, Cany Precision Instruments Co., Ltd., Shanghai, China). The SRF was calculated as follows:


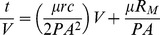


where *V* is volume of filtrate, *t* is time of filtration, *P* is pressure across filter medium and sludge, *A* is surface area of filtration, *μ* is filtrate dynamic viscosity, *r* is specific resistance to filtration, *c* is specific gravity, and *R_M_* is initial resistance of the filter medium. The equation is the commonly used linearized version(*y*  =  *k*x + *b*). The *r* can be calculated from slope *k* of a plot of t/V vs. V.

The water content, volatile suspended solids (VSS) and mixed liquor suspended solids (MLSS) were determined gravimetrically using the standard methods [29]. The sludge surface charge was assayed by colloidal titration [30]. Sludge EPS was extracted by first heating a sample [31]. The total carbohydrate composition of EPS was assayed by the phenol-sulfuric acid method [32]. Protein and humus levels were measured by the modified Lowry method [33]. Total organic carbon (TOC) was assayed by catalytic oxidimetry (Shimadzu TOC-VCPH) and used to determine the total EPS.
